# Supplementary material for: Syndecan-3 regulates MSC adhesion, ERK and AKT signalling in vitro and its deletion enhances MSC efficacy in a model of inflammatory arthritis in vivo
Source: Sci Rep. 2020 Nov 24;10:20487. doi: 10.1038/s41598-020-77514-z (PMC7686503; doi:10.1038/s41598-020-77514-z)
Supplement: Supplementary file 1 — Supplementary Figures. [file 41598_2020_77514_MOESM1_ESM.pdf]

Supplementary Information

Title: Syndecan-3 regulates MSC adhesion, ERK and AKT signalling in vitro and its deletion enhances MSC efficacy in a model of inflammatory arthritis in vivo

Authors: Fiona K Jones[1]†, Andrei Stefan[2]†, Alasdair G Kay[3], Mairead Hyland[2], Rebecca Morgan[2],

Nicholas R Forsyth[4], Addolorata Pisconti[1] and Oksana Kehoe[2]*

Supplementary Figures

Supplementary Figure 1. The following images are the full-length immunoblots from Figure 3. Black boxes indicate border of the blots.

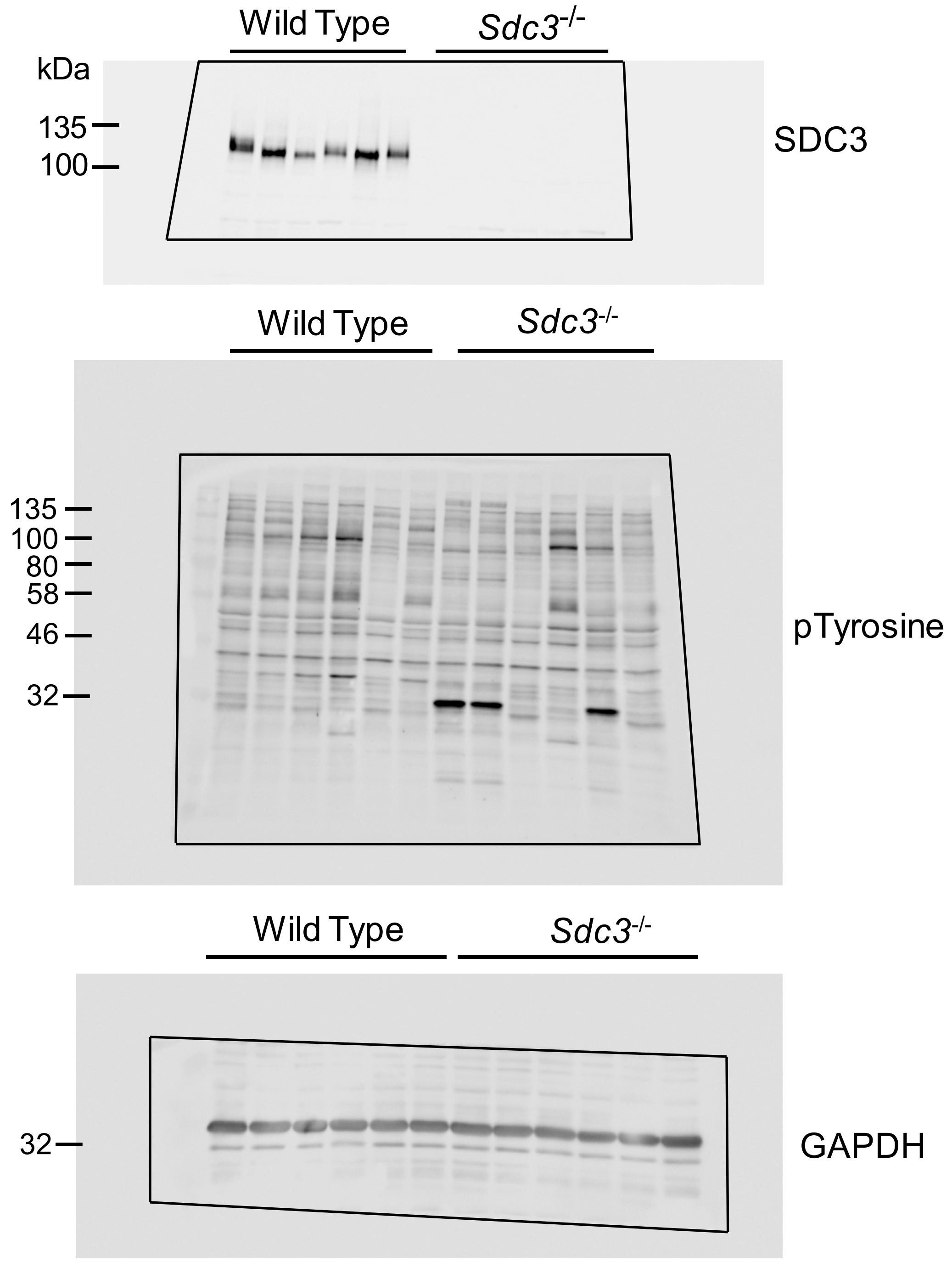

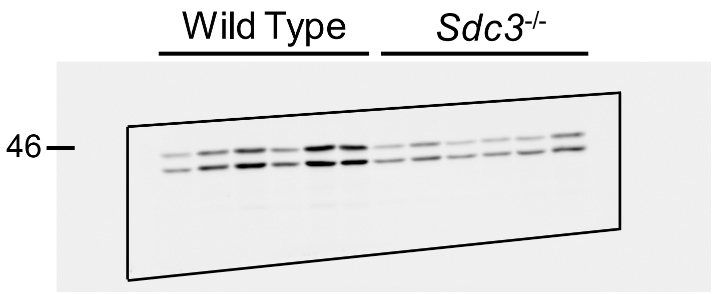

p-ERK

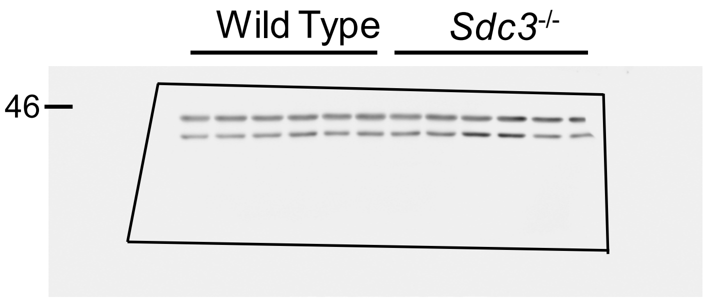

ERK

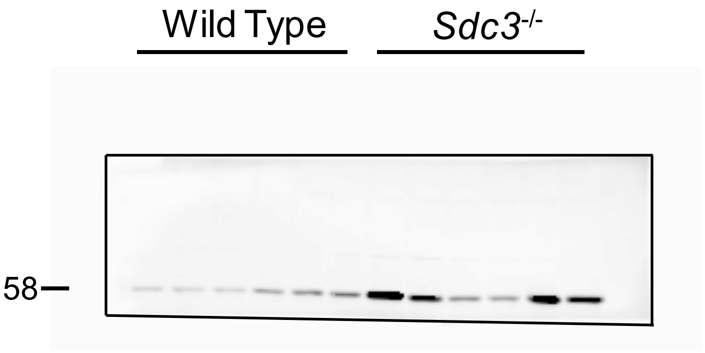

p-AKT

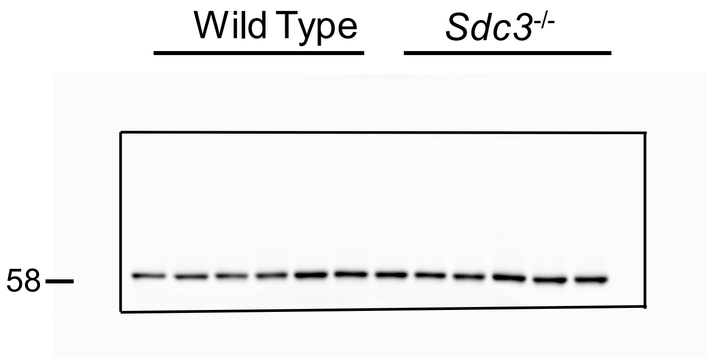

AKT

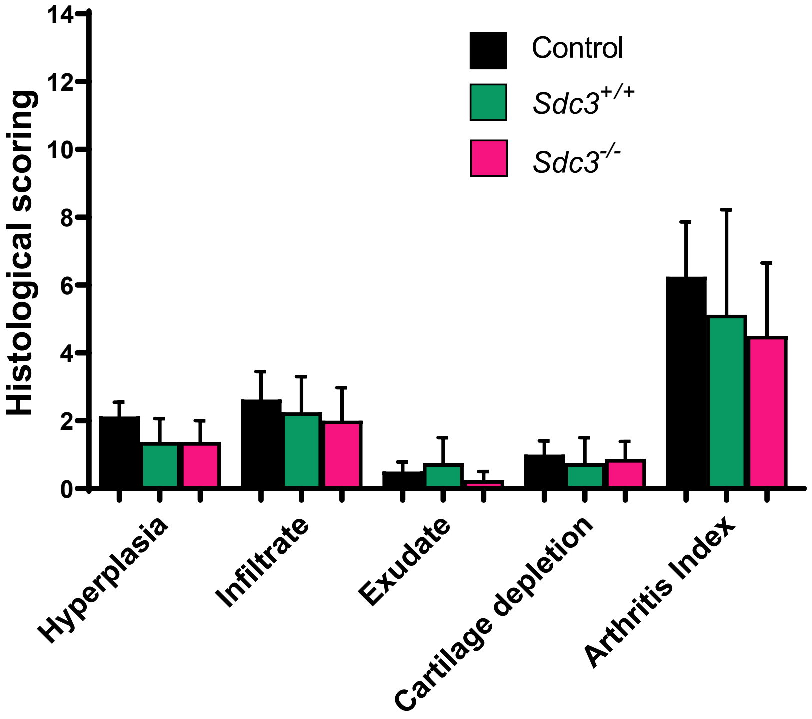

Supplementary Figure 2. The histological scoring for 7 days post arthritis induction. The arthritis index is the sum of all observations. Data are means ± SEM, n=4 mice in each group.
